# Retraction: Integrating clinical features, inflammatory markers, and immune profiles: a Yunke-based nomogram model for rheumatoid arthritis prognosis

**DOI:** 10.3389/fmed.2026.1867517

**Published:** 2026-05-04

**Authors:** Frontiers Editorial Office

The journal retracts the 30 June 2025 article cited above.

Following publication, concerns were raised regarding the validity of the data in the article. The authors failed to provide a satisfactory explanation during the investigation, which was conducted in accordance with Frontiers' policies.

This retraction was approved by the Chief Editors of Frontiers in Medicine and the Chief Executive Editor of Frontiers. The authors received a communication regarding the retraction and had a chance to respond. This communication has been recorded by the publisher.

